# Approximate Number Processing Skills Contribute to Decision Making Under Objective Risk: Interactions With Executive Functions and Objective Numeracy

**DOI:** 10.3389/fpsyg.2018.01202

**Published:** 2018-07-13

**Authors:** Silke M. Mueller, Matthias Brand

**Affiliations:** ^1^General Psychology: Cognition and Center for Behavioral Addiction Research, University of Duisburg-Essen, Duisburg, Germany; ^2^Erwin L. Hahn Institute for Magnetic Resonance Imaging, Essen, Germany

**Keywords:** decision making under risk, approximate number system, number processing, executive functions, numeracy, GDT

## Abstract

Research on the cognitive abilities involved in decision making has shown that, under objective risk conditions (i.e., when explicit information about possible outcomes and risks is available), superior decisions are especially predicted by executive functions and exact number processing skills, also referred to as objective numeracy. So far, decision-making research has mainly focused on exact number processing skills, such as performing calculations or transformations of symbolic numbers. There is evidence that such exact numeric skills are based on approximate number processing (ANP) skills, which enable quick and accurate processing of non-symbolic numbers (e.g., Chen and Li, 2014). Very few studies, however, have investigated ANP skills in the context of risky decision making and have analyzed direct associations among the aforementioned sub functions. Possible interactions between the closely related skills have not been considered. The current study (*N* = 128) examines interactions of ANP skills with executive functions and objective numeracy, in predicting risky choice behavior. ANP skills are represented by the accuracy in a dot-comparison task. Decision making is measured by two versions of the Game of Dice Task (GDT), which place different emphases on the reflection of potential risks. The results show two-way as well as three-way interactions between the measures of ANP skills, executive functions, and objective numeracy in predicting risky decisions in both GDT versions. The riskiest decisions were most frequently made in case of low scores in all of the three competencies, while good performance in any one of them resulted in significant reductions of disadvantageous decisions. The findings indicate that high ANP skills can positively affect choice behavior in individuals who have weaknesses in reflectively attributed skills, namely executive functions and objective numeracy. Potential compensatory effects and mechanisms of ANP in decision making are discussed.

## Introduction

Processing numbers is essential for many kinds of decisions. Numerical information can be useful in order to compare different options, to evaluate chances and risks, or to assess expected outcomes prior to a decision. However, numbers can be present in various contexts (e.g., finance or health risks), forms, and abstractions levels, e.g., as fractions (¼), percentages (25%), non-symbolic quantities (⋅/::), or absolute values (30 in 120). These different types of numerical information can sometimes be difficult to handle or to compare, because their interpretation requires different processing steps. Examples of different skills required for understanding numerical information are having knowledge about symbolic representations of numbers (that ‘4’ equals ‘::’), relating numbers to one another (that 120 is four times 30), or transforming different numeric information in order to compare both (e.g., to state whether ⅕ is larger or smaller than 0.25). These abilities are often summarized under the term ‘numeracy’ (Peters et al., 2006; Reyna et al., 2009). Studies have shown that even highly educated professionals have deficits in numeracy and thus lack understanding probabilistic numeric information (Estrada et al., 1999; Hoffrage et al., 2000; Lipkus et al., 2001). Research on the positive impact of numeracy on decision making (see Reyna et al., 2009; Peters, 2012; Schiebener and Brand, 2015 for comprehensive reviews) mainly focuses on the assessment of formal mathematical competence regarding symbolic numbers, such as basic calculation skills, and comprehension of different ratio formats as described above. However, recent theories and results of neurocognitive studies promote a differentiation between exact and approximate number processing (Piazza et al., 2004; Pica et al., 2004; Reyna and Brainerd, 2011). Further, a recent meta-analysis provides evidence that symbolic and non-symbolic numbers are not only processed by the same but also by distinct brain regions (Sokolowski et al., 2017). However, the two competencies seem to be associated with one another (see Chen and Li, 2014 for a review). So far, research on decision making under risk has focused on the involvement of (symbolic) exact number processing skills, while the role of basic (non-symbolic) approximate number processing skills has rarely been investigated. The current study aims to fill this gap by focusing approximate number processing skills, more precisely approximate non-symbolic number discrimination precision, and their potential interactions with numeracy and executive functions in the context of decision making under objective risk.

### Numeracy and Executive Functions in Decision Making Under Risk

Numeracy is generally defined as the ability to understand and use basic probabilistic information and numerical concepts (Peters et al., 2006; Reyna et al., 2009). The term numeracy is sometimes used as an umbrella term covering different types of numerical competencies (Peters and Bjalkebring, 2015). Typical objective measures of numeracy focus on the processing of probabilistic information (see Schwartz et al., 1997; Lipkus et al., 2001; Peters et al., 2006; for a review of different numeracy measures see Cokely et al., 2014). In such tasks, subjects are asked to make ordinal judgments of risk (e.g., 1 in 10 represents a bigger risk than 1 in 100 or 1 in 1000), to convert frequencies into percentages or vice versa (e.g., 10% risk is the same as 100 in 1000), or to demonstrate knowledge about basic principles of probabilities (e.g., that the chance of tossing a 1 or 6 using a normal six-sided die is twice as high as tossing a 3). In the following, for reasons of clarity and consistency, we will use the term ‘objective numeracy’ for the ability to accurately handle probabilistic numeric information in terms of exact symbolic numbers, as measured by tests as described above (e.g., the task by Lipkus et al., 2001).

Objective numeracy was found to have a significant impact on risk perception and medical decisions (e.g., Lipkus et al., 2010; for reviews see Reyna and Brainerd, 2007; Reyna et al., 2009) as well as on decision making in general, even when controlling for measures of general intelligence (Peters et al., 2006, 2007; Peters, 2012). Convergent results, obtained by use of laboratory gambling tasks, such as the Game of Dice Task (GDT; Brand et al., 2005), let assume that objective numeracy plays a key role in decision making under objective risk conditions, i.e., when the decision situation provides explicit information about possible outcomes and their probabilities (Schiebener and Brand, 2015). In the GDT, participants bet on the results of die-rolls by choosing either a single digit, or combinations of two, three, or four digits simultaneously. Options containing less digits are associated with higher gains but also with higher losses (single digit: ±1000; two digits: ±500; three digits: ±200; four digits: ±100), all given explicitly. In the GDT-Double, participants are also asked whether they want to double the amount of their bet or not.

Executive functions were shown to correlate with performance in the GDT and GDT-Double (Brand et al., 2005, 2006, 2007, 2014) as well as with performance in other tasks representing decision situations under objective risk conditions, such as the Columbia Card Task (Figner et al., 2009), and the Probability-Associated Gambling Task (Schiebener et al., 2011). In these tasks executive functions are assumed to enable the categorization of given information as well as strategy application and feedback inclusion. That is why models of decision making under objective risk consider executive functions to be a central predictor of advantageous choice behavior (Brand et al., 2006; Schiebener and Brand, 2015).

Linking numeracy and executive functions, it was found that ‘processing probabilities’ (measured by tests of probability knowledge and objective numeracy as mentioned above) mediate the effect of executive functions (Brand et al., 2014, Study 2) and mental calculation skills (Brand et al., 2014, Study 1) on the decision-making performance in the GDT-Double. Basic calculation skills are supposed to be relevant, e.g., for calculating expected values and thus for comparing different attributes and evaluating risks in a normative way (e.g., Cokely and Kelley, 2009; Brand et al., 2014). This assumption has recently been supported by Pertl et al. (2017), who showed that individuals with above average mathematical competence perform significantly better in the GDT-Double than average math performers. Thus, objective numeracy skills and executive functions act together in the process of decision making under objective risk.

However, besides careful consideration of the given (numeric) facts, decision makers also need to be able to extract and comprehend the meaning that underlies those facts, which is assumed to be another type of processing (Reyna and Brainerd, 1995; Lipkus and Peters, 2009). There is evidence that this ability might be even more relevant than exact numeric skills. For example, Cokely and Kelley (2009) showed by verbal protocol analysis that most decisions that were consistent with expected values did not originate from expected-value calculations, but from much simpler (nevertheless elaborative) considerations. It is further assumed that individuals with high objective numeracy were more inclined to extract and integrate the very essence from the given numeric information when making decision, which contributes to more accurate choice behavior (Cokely and Kelley, 2009). Accordingly, Peters et al. (2006) showed that individuals with higher objective numeracy could derive more affective meaning from numeric information than less numerate individuals. However, the tendency of people with higher numeric abilities to make more use of numeric information can sometimes lead to an overuse of exact numbers resulting in non-optimal evaluations (Peters et al., 2006; Kleber et al., 2013, Study 4).

### Approximate Number Processing

Besides numeracy, which concerns the processing of exact numbers, there is strong evidence that humans (and also other non-human species) have an innate ability to represent quantities of objects (i.e., non-symbolic numbers larger than four to five items) in an inexact or approximate way as vague mental magnitudes (Dehaene, 1997; Feigenson et al., 2004). The so-called Approximate Number System is supposed to enable rough but quick estimations and comparisons between such non-symbolic numbers. While some researchers assume other systems than the Approximate Number System to be responsible for the quick processing of non-symbolic numbers (e.g., Gebuis et al., 2016), there is a consensus on the general functionality of our brain to quickly process and compare non-symbolic numbers, or, as we will call it from now on, approximate number processing (ANP). The ANP precision is defined as the degree to which the quantities of two sets of items can be discriminated (e.g., Park and Brannon, 2013). A commonly used and recommended way to measure ANP precision is to use non-symbolic number-comparison tasks, such as dot-comparison tasks (see Dietrich et al., 2015). Dot-comparison tasks ask participants to indicate which of two shortly presented sets of dots was of higher quantity. Thereby, one can observe distance effects (see Moyer and Landauer, 1967), in a way that the closer the distance between the to-be-compared quantities, the more difficult they are to distinguish as indicated by increasing response time and decreasing accuracy. Hence, ANP precision (i.e., accuracy) is ratio dependent. Further, ANP precision increases from infancy to adulthood (Xu and Spelke, 2000; Halberda and Feigenson, 2008; Libertus and Brannon, 2010; Halberda et al., 2012). While two quantities with a ratio of 1:2 (e.g., 16 versus 32 dots) can be easily distinguished by adults, and even by 6-months old infants (Xu and Spelke, 2000), the individual differences in adults’ ANP precision significantly increase with ratios of about 9:10, e.g., 36 versus 40 dots (Halberda and Feigenson, 2008). Dot comparison tasks exist in many different versions (see Dietrich et al., 2015). Thereby the to-be-compared sets of dots are presented either sequentially, or simultaneously as separate pairs, or simultaneously intermixed, e.g., in different colors. The versions correlate only mildly to moderately, potentially due to differential demands of the tasks regarding, e.g., working memory, inhibitory control, or visual resolution (Price et al., 2012; Gilmore et al., 2014).

Although ANP skills and objective numeracy are substantively different, research suggests that both abilities are related to one another. Having high ANP precision, i.e., being good at discriminating even close-together quantities, is assumed to be a pre-stage of objective numeracy (see also Peters and Bjalkebring, 2015). There is growing evidence that ANP (or the respective underlying system) seems to build the cognitive basis for basic symbolic arithmetic (Gilmore et al., 2007; DeWind and Brannon, 2012; Feigenson et al., 2013; Libertus et al., 2013; Park and Brannon, 2013; Chen and Li, 2014) and potentially even for higher mathematics (Butterworth, 2005; Matthews et al., 2016). However, there are also some inconsistent findings regarding the link between ANP precision measured by dot-comparison tasks and measures of symbolic numeric competence (see De Smedt et al., 2013 for a review). These inconsistencies may result from the different methods used to measure ANP precision, which are partly of low reliability (Lindskog et al., 2013). The current study used a paired dot-comparison task. Dietrich et al. (2015) recommend the use of these kinds of tasks for measuring ANP precision, because of their frequent usage, the consistent correlations with other ANP tasks, and the low involvement of other cognitive processes.

In the context of decision making, research relating ANP precision with performance on decision-making tasks is still rare (see Winman et al., 2014). Regarding symbolic number mapping, there is first evidence suggesting that ANP skills play a distinct role. Studies by Peters and colleagues demonstrate that the precision of mental-number-line representations (e.g., symbolic number mapping) influences numerical reasoning and the valuation of decision options, while it was separable from objective numeracy (Peters et al., 2008; Schley and Peters, 2014; Peters and Bjalkebring, 2015). Such mapping skills were further assumed to have a compensatory effect for individuals who lack the time, motivation, or capacity to perform formal number calculations (Peters and Bjalkebring, 2015). Non-symbolic number processing has been studied in the context of probability judgments. For example, Winman et al. (2014) demonstrated that subjects with high ANP precision (measured by a non-symbolic number-comparison task) made more realistic judgments about their own performance. However, ANP precision, in contrast to objective numeracy, did not predict the calibration of probability judgments. Similarly, Patalano et al. (2015) demonstrated that objective numeracy but not ANP precision was associated with number distortions in a gambling task. Both studies used the number-comparison task by Halberda et al. (2008), which presents the to-be-discriminated stimuli in an intermixed format (i.e., blue and yellow dots).

In a recent study (Mueller et al., 2018), using a paired dot-comparison task, we did not find any direct association between ANP precision and the decision-making performance under objective risk measured with the GDT-Double. Rather, more integrative skills, such as being able to make accurate risk estimations from approximate number comparisons, predicted decision making in addition to the effects of executive functions and numeracy. What has not yet been investigated systematically, is the possible interplay between non-symbolic number processing skills (ANP precision), symbolic numeric skills (objective numeracy) and executive functions. The latter two have already been shown to constantly predict the decision-making behavior under objective risk conditions. However, it is unclear whether ANP skills (although probably not directly related) moderate these effects.

### Objectives and Hypotheses of the Study

The above mentioned findings from decision-making and numerical-cognition research let assume that numeric information can be processed, on the one hand, in an exact manner, and, on the other hand, inexactly or approximately as rough mental representations of numerosities (Piazza et al., 2004; Pica et al., 2004; Reyna and Brainerd, 2011). Irrespective of which processing mode may comprise which aspect of numerical information processing (for different dual-processing accounts of decision making see Reyna and Brainerd, 1995, 2011; Sloman, 1996; Stanovich and West, 2000; Schiebener and Brand, 2015) it is reasonable that, also in decision making, numbers and quantities are not only processed in an exact analytical way, but also more approximately in terms of rough estimations or other rather simple considerations. This assumption can be transferred to complex decision-making situations. For example, the most optimal decisions in the GDT are those for combinations of four digits as possible die-roll results, rather than choosing combinations of three or two digits, or a single digit. Optimal decisions in the GDT can potentially derive from different considerations: For example, one can calculate the chances and risks of the different decision options exactly (⅙ vs. ⅚; 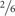 vs. 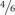; 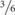 vs. 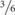; 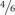 vs. 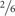) in order to compare them or to further calculate expected values for each option by additionally taking the possible gains/losses into account (e.g., 1000 × [⅙ - ⅚] vs. 100 × [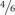 - 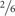]). On the other hand, one can also simply consider that betting on more digits simultaneously is less risky than betting on few digits, and, as a result, choose the options with the highest quantity of digits. Both strategies would lead to the same (optimal) decision, i.e., choosing the most advantageous option. However, in the latter case, the individual could have come to this decision without having performed any calculations and without having considered any exact number or quantity.

The aim of the current study was to investigate interactions of ANP skills with objective numeracy and executive functions. Objective numeracy, as an exact numeric processing skill, and executive functions have already been shown to (interactively) predict decision making under objective risk. Non-symbolic ANP skills were shown to be linked to numeracy. Based on previous findings, no direct effect of ANP skills on probability judgments and decisions could be expected. However, we assumed that high ANP precision may have a compensatory effect, as we suggested that advantageous decisions under risk can also result from the integration of non-exact considerations involving ANP, e.g., quick comparisons of item quantities representing risk information without the need for processing exact symbolic numbers. Thus, the current study examined ANP skills as the precision with which non-symbolic numbers (i.e., numerosities) can be discriminated.

We hypothesized interaction effects between ANP skills and executive functions as well as between ANP skills and objective numeracy in predicting risky decision making in the GDT. Furthermore, it can be assumed that ANP is particularly relevant when reflective processing skills are made less use of (or are rarely available) during decision making. Thus, we hypothesized three-way interactions between executive functions, numeracy, and ANP skills in predicting GDT decision making.

The current study used two slightly different versions of the GDT as measures of decision making under objective risk. The group variable (GDT version) served as between-subject factor in order to examine whether the hypothesized interactions apply for standard decision situations under objective risk (original GDT) as well as for objective risk situations that especially challenge reflection about risks (GDT-Double). Moreover, equal variables of both GDT versions allowed combined data analyzes across the whole sample.

## Materials and Methods

### Study Sample

The sample analyzed in the current study consisted of a total of 128 subjects (73 females, 55 males), aged from 18 to 62 years (*M* = 28.56, *SD* = 12.12). The sample comprised two groups of participants. One group (*n* = 64; 36 females, 28 males) performed the original version of the GDT as well as the other tasks mentioned below. This group stemmed from a new streamlined data collection. The other group (*n* = 64; 37 females, 27 males) consisted of age- and gender-matched participants from the sample reported in Mueller et al. (2018), which performed the GDT-Double instead of the original GDT as well as other tasks including the ones described below. Neither group included individuals with neurological or psychiatric diseases, as determined by a screening questionnaire. The participants were recruited at the University of Duisburg-Essen and by open recruitment. The experiment was approved by the ethics committee of the department of Computer Science and Applied Cognitive Science at the Faculty of Engineering at the University of Duisburg-Essen (Germany). All subjects gave written informed consent in accordance with the Declaration of Helsinki and were standardly debriefed afterwards. Student subjects were remunerated with credit points.

### Instruments

Each of the subjects performed the tests individually under similar controlled laboratory conditions. All of the computerized tasks were conducted in random order at the same computer screen per data collection. Questionnaires assessing sociodemographic and other variables not relevant for this study, were filled out in the breaks between two computer tasks. Participants were free to choose their preferred distance from the screen. The instructions for each of the tests were given in a standardized manner.

#### Number-Comparison Task

We used the first subtask of the Risk Approximation Task (RAT; Mueller et al., 2018) as a measure of ANP skills. The RAT number-comparison subtask depicts a paired dot-comparison task which is performed on a computer. The task was developed according to the recommendations for measuring ANP precision by Dietrich et al. (2015). It consists of 30 trials, in each of which paired arrays of white dots on dark gray background are simultaneously flashed on the computer screen for 300 ms. After each presentation, subjects were asked to indicate which of the two arrays contained more dots by pressing the left or right arrow key. Participants got visual feedback about the registered response, but not about its correctness. The dot arrays varied in numerosity (between 4 and 48 dots per array), ratio (0.25, 0.50, 0.75, 0.83, and 0.90; six trials each), and visual properties (e.g., subtended area, density, average diameter etc.) as generated with the software by Gebuis and Reynvoet (2011). The trials were presented in random order. Each stimulus pair was used twice to counterbalance the side on which the array with more dots is presented. Thereby, the stimulus pictures were rotated and/or mirrored when presented for the second time, in order to avoid recognition effects but to keep the visual properties stable. Accuracy scores (percent correct responses) served as performance measures. Accuracy scores were calculated across all trials as well as individually for each ratio. Scores closer to one indicate more precise ANP.

#### Modified Card Sorting Test (MCST)

The Modified Card Sorting Test (MCST; Nelson, 1976) served as a measure of executive functions, especially categorization, set-shifting, and feedback processing. The MCST is a computerized task that asks subjects to sort cards depicting symbols to one of four decks, according to specific rules. The to-be-sorted cards are presented separately with symbols that vary in shape, color, and quantity. By pressing button ‘1’ to ‘4’ the participant sorts the respective card to one of the four decks. The participant then receives auditory and visual feedback about whether the card was sorted correctly given the current rule (shape, color, or quantity). Once sorted correctly, the current rule must be followed until a message appears informing that the rule has changed. Errors can occur due to disregard of the current rule (i.e., sorting according to another rule than before although the rule has not changed) or errors can be due to perseveration (i.e., sticking with the prior rule although the rule has changed). The total number of errors (perseverative + non-perseverative errors) is used as a measure of executive functions with higher scores representing weaker executive functions.

#### Numeracy Task

The numeracy task, which was also used by Delazer et al. (2013), consists of 12 questions regarding probabilistic numbers, similar to the task by Lipkus et al. (2001). An example question is ‘Which of the following numbers represents the highest risk of getting a disease? 1 in 100, 1 in 1000, or 1 in 10?’ The questions were presented one by one on a computer screen. Subjects had to respond orally within 45 s per question. The sum of correct responses serves as a measure of objective numeracy (the higher the better).

#### Game of Dice Task (GDT)

In the current study, two versions of the computerized GDT were used. One group of subjects (*n* = 64) performed the original GDT. The other group (*n* = 64) performed the GDT-Double, which is a more demanding version that places more emphases on the processing of risks and ratios as described below.

##### GDT original

The GDT (Brand et al., 2005) is a computer task where subjects shall maximize a virtual capital of €1000 by betting on the results of multiple die rolls. In each of the 36 rounds, the subject can either bet on a single digit or on different combinations of digits, which are associated with different amounts of gains or losses. Participants place their bet by clicking on the respective digit (e.g., ‘5’) or combination of digits (e.g., ‘1 2 3 4’). Betting on a single digit reveals the highest gain/loss amount, but the lowest winning probability. Bets on combinations of two, three, or four digits are linked to, respectively, lower gain/loss amounts. Overall, the decision for one single digit is the riskiest choice (winning probability of ⅙) and the one with the most negative expected outcome. Conversely, choosing a combination of four digits is most advantageous in the long run because of a winning probability of more than 50% and a positive expected value (see **Table 1** for exact winning chances, gain/loss amounts, and expected values). The probabilities and amounts of gains/losses stay stable and are constantly displayed on the screen during the entire game. After each decision, the subjects receive explicit feedback about the respective outcome (gain/loss) and the actual account balance.

**Table 1:** Winning chances, gains/losses, and expected values of each type of decision option for the GDT and the GDT-Double.

		GDT (original)		GDT-Double (if doubled)	
Options	Winning chance (%)	Gain/Loss	EV	Gain/Loss	EV
One single digit	16.67	1000	–666.67	2000	–1333.33
Two digits	33.33	500	–166.67	1000	–333.33
Three digits	50	200	0	400	0
Four digits	66.67	100	33.33	200	66.67

*GDT, Game of Dice Task; in case the bet is not doubled in the GDT-Double, the gain/loss amounts and expected values (EV) of each option are the same as in the original GDT.*

##### GDT-double

The GDT-Double (for a more detailed description see Brand et al., 2014) is based on the same game principle as the original GDT. The only difference is that, in the GDT-Double, after each decision and before the result is shown the subject is asked whether the bet amount should be doubled or not. Thus, the GDT-Double is more complex compared to the original version, because additional outcomes can be expected (see **Table 1**). In contrast to the original version, the most advantageous option in the GDT-Double is not the most conservative one (without doubling), but betting on a combination of four digits and then doubling the amount (see **Table 1** for the expected values of the GDT and GDT-Double). Furthermore, the questions following each choice (whether to double or not) encourage subjects to (again) think about ratios and potential risks, which potentially triggers more reflective processing (Schiebener and Brand, 2015). For both the GDT and the GDT-Double, the difference between the number of advantageous and disadvantageous decisions (net score) was calculated as well as the number of decisions for the riskiest alternatives (one single digit).

## Results

Statistical analyses were carried out with SPSS 24.0 (IBM SPSS Statistics, released 2016). Pearson product-moment correlation coefficients (Pearson’s *r*) were calculated to test for bivariate correlations, with *r* ≥ 0.1 indicating a small, *r* ≥ 0.3 indicating a medium, and *r* ≥ 0.5 indicating a large effect (Cohen, 1988). Multiple hierarchical moderated regression analyses were conducted in order to test the effects of different predictors and interactions of these on the dependent variable. All predictors were mean centered.

In the following, the descriptive statistics and correlations across all measures are reported separately for the two groups, in order to compare the results between groups. Furthermore, moderated regression analyses were conducted in order to test the aforementioned interaction hypotheses. Two blocks of analyses were run in order to test the first hypothesis. The third block of analyses tested the hypothesized three-way interaction between the three predictors, namely executive functions (MCST errors), objective numeracy (correct responses in the numeracy task), and ANP skills (accuracy in the number-comparison task).

### Descriptive Statistics

The groups (GDT original and GDT-Double) differed in none of the parameters, except the net score (see **Table 2**). Subjects who played the original GDT had higher net scores than those who played the GDT-Double, however, the mean GDT-Double net score was generally low compared to other studies (see Brand et al., 2014; Pertl et al., 2017). The groups did not differ in the mean number of riskiest decisions, i.e., choosing one single digit. Thus, this score was included in the following analyses as the measure of risky GDT decision making. The performance scores in the number-comparison task were also statistically equal for both groups (see **Table 2**). Overall, a typical ratio effect could be observed, i.e., the closer-together the to-be-compared quantities were, the poorer was the accuracy (ratio ¼: *M* = 0.988, *SD* = 0.048; ratio ½: *M* = 0.951, *SD* = 0.099; ratio ¾: *M* = 0.733, *SD* = 0.178; ratio ⅚: *M* = 0.723, *SD* = 0.165; ratio 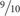: *M* = 0.565, *SD* = 0.178). Accordingly, the highest variability in discrimination precision across subjects was found for dot-arrays of ratio 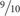. Accuracy at ratios ½ and ¼ were non-normally distributed with skewness of -4.38 (*SE* = 0.214) and -2.33 (*SE* = 0.214) indicating ceiling effects (Koedel and Betts, 2010). Thus, besides the mean overall accuracy, we additionally computed a mean score excluding the trials which produced ceiling effects (i.e., including trials with ratio ¾ or larger) in order to have an additional measure that is more sensitive to individual differences in ANP precision. Following this approach, we additionally took an even more sensitive score. As adults from typically educated cultures can discriminate quantities of ratios up to 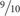 consistently (Pica et al., 2004; Halberda and Feigenson, 2008), we wanted to have a closer look at the accuracy at this more difficult ratio 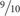 (i.e., the most difficult trials in the number-comparison task used in this study, *M* = 0.565, *SD* = 0.178). Thus, the following analyses were conducted on all three measures of ANP skills, respectively, namely number-comparison (1) total accuracy, (2) accuracy at ratios ≥ ¾, and (3) accuracy at ratio 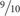.

**Table 2:** Descriptive statistics and comparisons of the main variables between groups.

			GDT original (*n* = 64)		GDT-double (*n* = 64)			
	Minimum	Maximum	*M*	(*SD*)	*M*	(*SD*)	*t*	*p*
Age	18	62	28.56	(12.50)	28.56	(11.82)	<0.01	>0.999
MCST (total errors)	0	32	8.91	(8.34)	7.23	(6.67)	1.25	0.213
Numeracy task (total correct)	1	12	10.08	(2.19)	10.03	(1.79)	0.13	0.895
Number-comparison task (accuracy)								
Total	0.600	0.930	0.793	(0.070)	0.791	(0.066)	0.13	0.897
Ratio ≥ ¾	0.389	0.889	0.674	(0.111)	0.674	(0.096)	<0.01	>0.999
Ratio 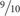	0	1	0.565	(0.194)	0.565	(0.162)	<0.01	>0.999
Game of Dice Task (GDT)								
Net score	–18	18	10.75	(9.29)	7.17	(10.46)	2.05	0.043
One single digit	0	18	1.19	(3.18)	1.59	(2.99)	–0.74	0.458

*MCST, Modified Card Sorting Test*.

### Correlation Analyses

The results of the correlation analysis (see **Table 3**) show that in both groups (i.e., both GDT versions) decision-making performance is associated in similar patterns with measures of executive functions (MCST), objective numeracy (numeracy task), and ANP precision (number-comparison task). The only exception was the correlation between measures of numeracy and ANP precision, which occurred only in the group that played the GDT-Double (see **Table 3**). As expected from previous findings, higher GDT performance (indicated by higher net score/lower ‘one single digit’) correlates negatively with weak MCST performance (indicated by the number of errors), and positively with correct responses in the numeracy task. There is no direct correlation between GDT performance and the accuracy in the number-comparison task in neither group.

**Table 3:** Bivariate correlations between measures of executive functions, numeracy, ANP precision, and decision making under risk, separately for the two GDT versions.

		GDT original (*n* = 64)				GDT-double (*n* = 64)			
	Task (score)	1	2	3	4	1	2	3	4
(1)	MCST (total errors)	–				–			
(2)	Numeracy (total correct)	–0.297*	–			–0.329**	–		
(3)	Number-comparison (total accuracy)	0.074	–0.065	–		–0.089	0.250*	–	
(4)	GDT (net score)	–0.269*	0.378**	0.007	–	–0.294*	0.201	–0.099	–
(5)	GDT (one single digit)	0.229	–0.637**	–0.053	–0.742**	0.308*	–0.306*	0.008	–0.671**

*GDT, Game of Dice Task; MCST = Modified Card Sorting Test; ^∗^p < 0.05, ^∗∗^p < 0.01.*

### Moderated Regression Analyses

In order to investigate the hypothesized interaction effects of the measures of ANP skills with measures of executive functions and objective numeracy, moderated regression analyses were performed. First, the interactions between the measures of executive functions and ANP abilities were analyzed. The group variable ‘GDT version’ was included as an additional predictor in order to control for group effects. Secondly, we tested interactions between numeracy and ANP abilities. Here again, the group variable was also included. Thirdly, we examined three-way interactions between the measures of executive functions, objective numeracy, and ANP skills in predicting overall GDT performance. All analyses were conducted with number-comparison ‘total accuracy’ as well as with the two more sensitive scores [i.e., ‘number-comparison (accuracy at ratios ≥ ¾)’ and ‘number-comparison (accuracy at ratio 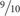)’].

#### Interactions Between Executive Functions and ANP Skills

The first hierarchical regression analysis included the group variable ‘GDT version’ in the first step, followed by ‘MCST’ (total errors) and ‘number-comparison’ (accuracy) in the second and third steps. Then, all two-way interaction terms and, lastly, the three-way interaction term were included. The number of choices for the riskiest alternative (GDT one single digit) was the dependent variable. The results of the analysis including ‘number-comparison (total accuracy)’ as ANP-skill measure show no significant interactions. MCST was the only predictor of GDT performance (for the model summary see **Appendix Table A1**).

The equivalent analysis with ‘number-comparison (accuracy at ratios ≥ ¾)’ reveals similar results as the previous analysis, but with one important difference: The interaction ‘MCST × number-comparison’ (step 6) significantly explains additional variance in the GDT score, Δ*R*^2^ = 0.044, Δ*F* = 6.19, *p* = 0.014. The three-way interaction with group is not significant, Δ*R*^2^ < 0.001, Δ*F* = 0.02, *p* = 0.888. Besides the interaction with number-comparison, the MCST is the only predictor with a significant coefficient in the final model, β = 0.316, *T* = 3.50, *p* = 0.001.

**FIGURE 1:**
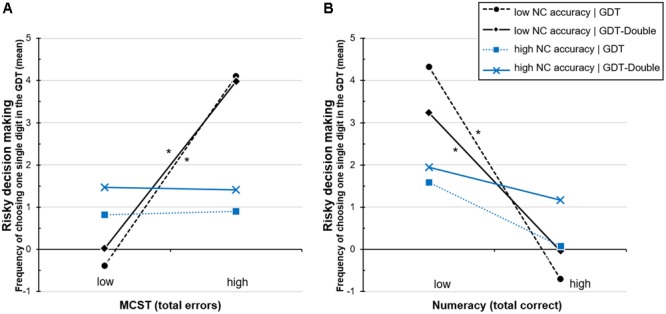
Simple slopes for the regression analyses testing interactions between **(A)** group (task version), executive functions (errors in the Modified Card Sorting Test; MCST), and ANP skills (number-comparison accuracy; NC accuracy) as well as between **(B)** group, objective numeracy, and ANP skills on risky decision making in the Game of Dice Task (GDT one single digit). The ‘high’ and ‘low’ data points represent values of 1 SD above/below the mean. ^∗^*p* < 0.05.

The results using ‘number-comparison (accuracy at ratio 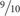)’ show that number-comparison still has no direct effect on GDT decisions, Δ*R*^2^ = 0.013, Δ*F* = 1.72, *p* = 0.192. As in the previous analyses, no interactions with the group variable are present, neither with MCST, Δ*R*^2^ = 0.002, Δ*F* = 0.33, *p* = 0.570, nor with number-comparison, Δ*R*^2^ = 0.001, Δ*F* = 0.19, *p* = 0.663, nor with both (three-way), Δ*R*^2^ < 0.001, Δ*F* = 0.03, *p* = 0.857. In contrast, adding the interaction term ‘MCST × number-comparison’ significantly explains additional 11% of the variance in ‘GDT one single digit’, Δ*R*^2^ = 0.110, Δ*F* = 16.55, *p* < 0.001. The final model accounts for almost 20% of the variation in GDT decision making, total *R*^2^ = 0.199, *F*(7,127) = 4.26, *p* < 0.001 (for the coefficients of the final model see **Appendix Table A2**). **Figure 1A** visualizes the interaction effect with regard to the two groups. For both groups, if number-comparison accuracy is high (see **Figure 1**, blue lines) the riskiest option (GDT one single digit) is barely chosen, irrespective of MCST performance. However, if number-comparison (accuracy at ratio 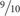) is low (see **Figure 1**, black lines), subjects of both groups make significantly more of the riskiest choices when they show weak rather than good MCST performance (weak performance is indicated by a high number of errors).

#### Interactions Between Numeracy and ANP Skills

The second analyses were similar to the first ones, with the difference that ‘numeracy’ (total correct responses in the numeracy task) was included instead of ‘MCST’. Results of the analysis including ‘number-comparison (total accuracy)’ show no significant predictors, except numeracy in the second step, which accounted for about 24% of the variance in GDT performance (for the model summary see **Appendix Table A3**).

Similarly, the same analysis with the ‘number-comparison (accuracy at ratios ≥ ¾)’ shows no significant interactions of number-comparison with numeracy, neither two-way (step 6), Δ*R*^2^ = 0.016, Δ*F* = 2.69, *p* = 0.103, nor three-way with group (step 7), Δ*R*^2^ = 0.008, Δ*F* = 21.38, *p* = 0.243.

However, performing the same analysis with the score ‘number-comparison (accuracy at ratio 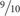)’ reveals significance for the interaction with numeracy, Δ*R*^2^ = 0.054, Δ*F* = 9.86, *p* = 0.002. As in the other analyses, the three-way interaction with the group variable causes no additional changes, Δ*R*^2^ = 0.002, Δ*F* = 0.29, *p* = 0.594. The final model accounts for one third of the variance in risky GDT decisions, total *R^2^* = 0.333, *F*(7,127) = 8.57, *p* < 0.001 (for the coefficients of the final model see **Appendix Table A4**). The mentioned interaction between numeracy and number-comparison (accuracy at ratio 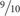) is visualized in **Figure 1B**. Similar to the previous analyses regarding interactions with executive functions (see section ‘Interactions Between Executive Functions and ANP Skills’), subjects with low numeracy show more of the riskiest GDT decisions than those with high numeracy. However, this difference is only significant in case number-comparison accuracy at ratio 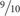 is additionally low (see **Figure 1**, black lines). Accordingly, in both GDT versions, ‘one single digit’ is chosen most frequently when performance in both the numeracy task and the number-comparison task are low. However, we like to note that the accuracy at ratio 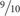 is based on only a few number of trials, which is why these results should to be treated with caution, as discussed later.

#### Interactions Between Executive Functions, Numeracy, and ANP Skills

The third analysis tested interaction effects between the measures of executive functions, objective numeracy, and ANP skills. Because ‘GDT version’ did not have any effect in the previous analyses (neither a direct effect, nor two-way or three-way interaction effects with the other predictors), the two groups were considered together for the following analyses. The number of choices for ‘one single digit’ in the GDT (both versions) was again the dependent variable. ‘MCST’ (total errors) was added as the first, ‘numeracy’ (total correct) as the second, and ‘number-comparison’ (accuracy) as the third predictor, followed by the respective interaction-terms in steps four to seven.

The results reveal that, in addition to the effect of MCST performance (Δ*R*^2^ = 0.064, Δ*F* = 8.59, *p* = 0.004), numeracy has a significant direct effect on ‘GDT one single digit’ (Δ*R*^2^ = 0.190, Δ*F* = 31.72, *p* < 0.001), while number-comparison accuracy has not, with any score (total accuracy: Δ*R*^2^ < 0.001, Δ*F* = 0.01, *p* = 0.928; accuracy at ratios ≥ ¾ : Δ*R*^2^ < 0.002, Δ*F* = 0.28, *p* = 0.596; accuracy at ratio 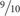 : Δ*R*^2^ = 0.014, Δ*F* = 2.40, *p* = 0.124). The interaction of ‘MCST × numeracy’ explains additional 12% of variance in GDT performance (in the analysis including number-comparison total accuracy: Δ*R*^2^ = 0.123, Δ*F* = 24.24, *p* < 0.001). The addition of the two-way interactions with number-comparison accuracy causes no changes when using the total accuracy score (‘MCST × number-comparison’: Δ*R*^2^ = 0.004, Δ*F* = 0.88, *p* = 0.351; ‘numeracy × number-comparison’: Δ*R*^2^ < 0.001, Δ*F* = 0.09, *p* = 0.764). The respective three-way interaction fails to reach significance, Δ*R*^2^ = 0.018, Δ*F* = 3.62, *p* = 0.060.

Interestingly, looking at the results of the same analysis with the two more sensitive number-comparison scores reveals three-way interactions accounting for additional GDT variance. In detail, the interaction of ‘MCST × numeracy × number-comparison (accuracy at ratios ≥ ¾)’ explains additional 3.5%, Δ*F* = 7.36, *p* = 0.008, while the three-way interaction with the most sensitive ANP score (number-comparison accuracy at ratio 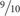) accounts for almost 4%, Δ*R*^2^ = 0.039, Δ*F* = 9.23, *p* = 0.003. In sum, the final models including all predictors account for, respectively, 43.5% [number-comparison accuracy at ratios ≥ ¾: *F*(7,127) = 13.20, *p* < 0.001] and 49.8% [number-comparison accuracy at ratio 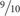 : *F*(7,127) = 16.98, *p* < 0.001] of ‘GDT one single digit’. The coefficients (see **Table 4**) show that MCST, numeracy, and the interaction of both have incremental effects on risky GDT decisions, with numeracy having the highest effect size. However, the three-way interaction effect is also of noticeable magnitude (see **Table 4** for the coefficients of the most predictive model).

**Table 4:** Statistics of the coefficients in the final step of the moderated regression analysis examining interaction effects between measures of executive functions (MCST), objective numeracy (numeracy task), and ANP skills (number-comparison accuracy) on risky decision making in the GDT (one single digit).

Model variables	*b*	*SE*	β	*t*	*p*
**Predictor variables**					
MCST (total errors)	0.06	0.03	0.144	2.00*	0.048
Numeracy task (total correct)	–0.46	0.11	–0.300	–4.07***	<0.001
Number-comparison task (accuracy at ratio 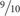	–0.29	1.21	–0.017	–0.24	0.813
**2-way interactions**					
MCST × Numeracy	–0.05	0.02	–0.258	–3.48***	≤0.001
MCST × Number-comparison	–0.33	0.17	–0.145	–1.95	0.053
Numeracy × Number-comparison	0.83	0.63	0.097	1.32	0.188
**3-way interaction**					
MCST × Numeracy × Number-comparison	0.22	0.07	0.240	3.04**	0.003

*GDT, Game of Dice Task; MCST, Modified Card Sorting Test; Please note that the positive beta value of MCST errors indicates an association between weak executive functions (more errors) and weak GDT performance (more risky choices). Negative beta values indicate a positive effect of the respective predictor (high value) on good GDT performance (less risky choices); ^∗^p < 0.05, ^∗∗^p < 0.01, ^∗∗∗^p < 0.001.*

**FIGURE 2:**
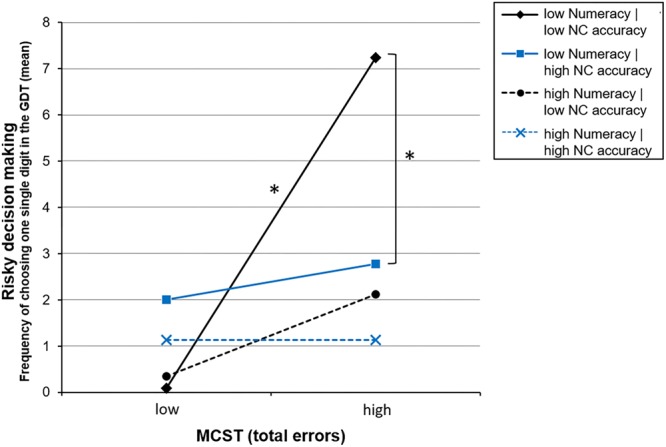
Simple slopes for the 3-way interaction between executive functions (errors in the Modified Card Sorting Test; MCST), objective numeracy (correct responses in the numeracy task), and ANP skills (number-comparison accuracy; NC accuracy) on risky decision making in the Game of Dice Task (GDT one single digit). The ‘high’ and ‘low’ data points represent values of 1 SD above/below the mean. ^∗^*p* < 0.05.

As visualized in **Figure 2** (left side), individuals with high executive functions (indicated by a low number of MCST errors) do not, or very rarely, choose the most disadvantageous options in the GDT (i.e., one single digit), irrespective of the extent of numeracy and number-comparison performance. Also, individuals with high numeracy scores make only few of the riskiest decisions (see **Figure 2**, dashed lines). For individuals with high numeracy, it can be observed that those with additionally weak MCST performance (high MCST errors) make more of the riskiest choices than those with good MCST performance (see **Figure 1**, black dashed line), however, this difference is not significant, *t* = 1.70, *p* = 0.092. Interestingly, individuals with weak performance in both the numeracy task and the MCST show very risky decision making (high choices of GDT one single digit), but only if accompanied by additionally low number-comparison accuracy (see **Figure 2**, black solid line). In contrast, individuals with high number-comparison accuracy (see **Figure 2**, blue solid line) on average choose ‘GDT one single digit’ significantly less frequent than those with low number-comparison accuracy, even if accompanied by weaknesses in numeracy and MCST performance, *t*(120) = 4.29, *p* < 0.001.

## Discussion

The current study aimed to investigate whether ANP skills contribute to decision making under objective risk conditions. More precisely, ANP skills (representing numerosity discrimination precision) were assumed to partly compensate for weaknesses in skills that promote the processing of exact numbers, namely executive functions and objective numeracy. ANP skills were measured with a number-comparison task using paired dot-arrays. The decision-making performance was assessed by the GDT and the GDT-Double in order to investigate whether the hypothesized interactions with ANP skills occur in both decision situations under risk, which emphasize either more (GDT-Double) or less (GDT original) reflecting on the riskiness of options. The analyses revealed two-way as well as three-way interactions between number-comparison accuracy at high ratios and the measures of executive functions (errors in the MCST) and objective numeracy (correct responses in a numeracy task), showing that ANP skills do not have a direct but can have an indirect compensatory effect on the decision-making performance under objective risk.

First, interactions between executive functions and ANP skills as well as, secondly, between numeracy and ANP skills were investigated within separate analyses. ANP skills were represented by three scores that differed in the difficulty of ratios included in the accuracy measure. The GDT version was taken into consideration by including the group variable as an additional predictor in both types of regression analyses. On the one hand, the results replicate previous findings (Brand et al., 2009, 2014; Schiebener et al., 2011; Pertl et al., 2017) by showing that executive functions and objective numeracy predict decision making in different versions of the GDT. The current results refer to those sub-components of executive functions that are associated with MCST performance, namely set-shifting, categorization, perseveration, and feedback processing (see Spreen and Strauss, 1998). Moreover, the findings further complement previous studies showing no direct association between ANP precision and decision making (Winman et al., 2014; Patalano et al., 2015; Mueller et al., 2018). On the other hand, the current results extend previous findings by indicating that ANP skills (at least when looking at the accuracy at more difficult to discriminate ratios) interacted with executive functions and (for accuracy at ratio 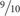) with numeracy in predicting decision making under objective risk (indicated by the number of riskiest choices in the GDT). The predictor ‘GDT version’ had no effect, neither directly nor in interactions with the other predictors. This indicates that the interaction between ANP skills and executive functions as well as the interaction between ANP skills and objective numeracy appear independent of whether the decision situation explicitly promotes reflection about the risks of given options (as in the GDT-Double) or not (as in the original GDT).

The third part of analyses combined both groups and investigated the three-way interaction effect between executive functions (a), objective numeracy (b), and ANP skills (c). The analysis with number-comparison accuracy including all ratios revealed no effects of ANP skills. However, looking at more sensitive scores revealed a significant three-way interaction between executive functions, numeracy, and ANP skills. This three-way interaction added explained variance to such explained by the single and two-way interactions. Besides the direct effect of numeracy (b) and the effect of its interaction with executive functions (a × b), the three-way interaction (a × b × c) was the third most influential predictor. The direct effect of executive functions (a) remained significant as well as (marginally) its interaction with ANP skills (a × c). The two-way interaction between numeracy and ANP skills (b × c) did not remain significant in consideration of the three-way interaction.

Looking at these interaction effects in more detail (for an illustration see **Figure 1**) reveals three main results: firstly, decision making under objective risk is mainly predicted by objective numeracy. High numeracy is associated with less disadvantageous decision making, even in individuals with weak executive functions and weak ANP skills. This underlines the importance of numeracy, described as the ability to handle (exact) probabilistic numbers and risk information, for making advantageous decisions under risk, which is in line with previous research and theoretical models (Reyna et al., 2009; Peters, 2012; Schiebener and Brand, 2015). Secondly, also executive functions contribute to making less of the very risky decisions. This is in line with previous findings showing that high executive functions measured by the MCST (i.e., being good at categorizing options, processing feedback, set-shifting, detecting rules, and pursuing a certain strategy) favor optimal decision making under objective risk conditions (Brand et al., 2007, 2009; Figner et al., 2009; Schiebener et al., 2011; Buelow, 2015). Thirdly, and most importantly for this study, the findings indicate that ANP skills can moderate the effects of numeracy and executive functions on the decision-making performance under objective risk. At least, this was the case for advanced ANP skills represented by the accuracy to discriminate ratios larger than ¾. Decision-making under objective risk seems to be susceptible to individual differences in ANP skills in case both numeracy and executive functions are low. This is indicated by the fact that individuals with low performance in each of the three domains are more prone to making disadvantageous decisions. However, low numerate individuals with low executive functions tend to make more of the most disadvantageous choices than high numerates only if accompanied by low ANP skills. Accordingly, less numerate individuals do not differ from high numerates regarding decisions under objective risk in case they are equipped with advanced ANP skills. Thus, ANP skills seem to be capable of at least partly compensating for limited abilities associated with analytical processing, namely executive functions and probability processing skills as assessed by objective numeracy tasks (see e.g., Schiebener and Brand, 2015). These findings do not contradict but rather add to previous studies on the involvement of ANP skills in judgment and decision making. In line with the findings by Winman et al. (2014), Patalano et al. (2015), and our previous study (Mueller et al., 2018), the current results do not indicate any direct associations between ANP skills and performance in a risky decision-making task. However, high ANP skills appear to come into effect when the capabilities for normative calculations and/or analytical processing of the given risk information are limited (i.e., when numeracy and executive functions are low). The current study supports this view at least for those subcomponents of executive functions related to set-shifting (Spreen and Strauss, 1998), while no conclusions can be drawn on other concepts of executive functions like e.g., working memory or inhibition (see Packwood et al., 2011). Similarly, the current results refer to those subcomponents of objective numeracy measured by Lipkus’ numeracy test, namely comparing, converting, and calculating with probabilistic numbers (Lipkus et al., 2001).

The results indicate that ANP precision, defined as the ability to quickly and accurately process numeric information in terms of numerosities (also referred to as ‘Number Sense’ or Approximate Number System accuracy, e.g., Libertus et al., 2013), has a positive impact on the decision-making performance under objective risk in case individuals lack in exact numeric skills and executive functions. Thus, a compensatory effect of ANP skills can be assumed. Similarly, Peters and Bjalkebring (2015) suggested a compensatory effect of number-mapping skills regarding individuals with a diminished capacity (or less time or motivation) to perform exact number operations. The precision of mental-number-line representations have previously been shown to effect the valuation of decision options and to be separable from objective numeracy (Peters et al., 2008; Schley and Peters, 2014; Peters and Bjalkebring, 2015). Other than symbolic-number mapping, ANP refers to non-symbolic numbers (i.e., numerosities) only (Gebuis et al., 2016). However, the current findings are consistent with the former, as both symbolic and non-symbolic approximation abilities share the processing of mental magnitude representations potentially involving the same cognitive system (Feigenson et al., 2004).

According to recent models, executive functions and probability processing are especially relevant for decision making under objective risk conditions (Schiebener and Brand, 2015) and would be attributed to reflective/analytical processing (see Sloman, 1996; Stanovich and West, 2000; Reyna and Brainerd, 2011; Schiebener and Brand, 2015). The results of the current study indicate that reflectively attributed skills (namely executive functions and objective numeracy) interact with quick inexact processing of (approximate) numbers. With regard to dual-process approaches, the moderating effects of ANP skills may be interpreted in different ways, which in turn need not to be mutually exclusive. Individuals with low executive functions and low numeracy are assumed to base their decisions on other than numeric information (Pertl et al., 2017) and are presumed to be more prone to impulsive than reflective processing (Schiebener and Brand, 2015). Thus, on the one hand, it could be argued that basic ANP comes in useful for non-deliberative decisions. Thereby, the ability to quickly and accurately discriminate even close-together sets of items (i.e., high ANP skills) may influence whereupon the impulsive decision is based in the respective decision situation. This beneficial effect of ANP might appear in a rather unaware manner. Some researchers argue that in some cases, unconscious cognitive processes are more beneficial than conscious ones, especially in complex decision-making situations (Dijksterhuis and Nordgren, 2006; Dijksterhuis et al., 2006). On the other hand, it could be argued that ANP skills benefit reflective considerations, especially when other skills that are involved in reflective processing, such as executive control functions and exact processing of numbers and ratios (Schiebener and Brand, 2015), cannot be referred to. For example, in the GDT, low numerate individuals can have deficits in calculating expected values or transferring the given information into exact risks (Brand et al., 2014; Pertl et al., 2017). Instead, one can base decisions on simple, heuristic-like considerations, such as ‘betting on more digits is better than betting on less digits’, which is an example of gistbased processing (e.g., see Reyna and Brainerd, 2011). Using this simple heuristic can result in optimal decision making in the GDT without having considered any exact number and without the imperative need of higher order executive functions, such as set-shifting or cognitive flexibility. Hence, high ANP skills may promote the integration of (simple concepts of) non-symbolic numeric information during the decision-making process. This integration potentially favors advantageous choice behavior, as superior decisions were shown to also originate from elaborative but rather simple, heuristic-like considerations as the one described above (Cokely and Kelley, 2009). However, the test of different dual-process approaches of decision making is beyond the scope of the present study.

In sum, the current findings confirm previous research suggesting no direct association between ANP skills and decision making (Winman et al., 2014; Patalano et al., 2015; Mueller et al., 2018), but furthermore provide new insights into the cognitive skills involved in decision making under objective risk. The results provide a first hint that exact analytical number processing and quick unlearned (but precise) processing of approximate numbers interactively determine decision making involving the consideration of numeric risk information, i.e., decisions under objective risk conditions. High ANP skills appear to be especially relevant when the capabilities for normative calculations and/or analytical processing of the given risk information are limited. Moreover, the results of the current study provide a first hint that ANP skills may act as a moderating factor. However, it remains unclear at which point exactly in the decision-making process ANP skills come into effect.

The current findings should be treated with caution due to limitations of the ANP measure. Compared to other non-symbolic number-comparison tasks, the number of 30 trials is much lower, which reduces its reliability (see Lindskog et al., 2013). The short amount of trials makes the task practicable in terms of using it in complex settings to avoid fatigue effects due to long lasting testing procedures. With respect to the general discussion about methodological issues when measuring ANP precision (see Gilmore et al., 2011), we encourage future studies to test the retest reliability of the current measure as well as to use other convergent measures for investigating the role of ANP skills in decision making under risk. A further limitation to be noted is that the positive impact of ANP abilities on decision making under risk conditions (irrespective of being involved in either impulsive or reflective processing or both) is, as often, probably dependent on the modalities and characteristics of the decision-making situation. In decision situations different from GDT decisions, it could be misleading or even disadvantageous to focus on the quantities of presented items. Whether the positive impact of ANP skills is then weakened or even reversed should be subject of future investigations. Further evidence is necessary to make meaningful conclusions on the impact of ANP abilities and their interactions with other cognitive functions.

Further, future studies should investigate whether the findings of this examination with healthy subjects also apply to patients with specific cognitive impairments and to older individuals. Aging as well as neurocognitive diseases are often accompanied by reductions in executive functions (e.g., Buckner, 2004; Jurado and Rosselli, 2007). As ANP skills were shown to remain unaffected by aging (Norris et al., 2015), the preservation and training of ANP skills (DeWind and Brannon, 2012; but see Szücs and Myers, 2017) might be of particular relevance for both interest groups in order to compensate for impaired executive functions and thus to prevent from very risky decision making with potentially serious negative implications.

## Conclusion

The results of the current study suggest that some individuals can profit from precise ANP when making decisions that provide objective information about risks. Although the data should be interpreted cautiously, the results provide a first hint that high ANP skills may play an important compensatory role, although they do not directly predict decision-making performance. More precisely, accurate ANP appears to especially benefit individuals with reductions in executive functions and/or reductions in exact ratio processing skills. Thus, individual differences in ANP potentially come into effect in decision making, when the skills that are relevant for formal analytical processing are rarely available. Under such circumstances, an accurate (normative) evaluation of chances and risks in terms of exact values is impeded. Then, however, the ability to quickly and accurately compare different amounts of items (i.e., high ANP skills) seems to be capable of preventing from very risky choices. This mechanism may not be limited to decisions in the GDT. It can be assumed to occur in various other decision-making situations, in which beneficial choices can be made on the basis of simple inexact number considerations (e.g., ‘Where are more?’), rather than on calculative operations on exact numbers (e.g., expected value calculations) only. However, it is likely that the reported effects do not consistently appear in other tasks, as the involvement of non-symbolic ANP skills in decision making under risk probably depends on the constitution of the specific choice scenarios. Nevertheless, we assume interactive effects of ANP skills with other cognitive functions to positively influence decision making in situations under objective risk, in which at least some part of the relevant numeric information is represented non-symbolically. More research is necessary to shed light on the mechanisms and the extent to which approximate number skills influence decision making, both directly and indirectly by potentially compensating for deficits in other individual competencies. Thus, ANP skills might be assumed to be of particular importance in order to counter serious decision-making deficits due to age-related and/or disease-related reductions in executive or other cognitive functions.

## Author Contributions

SM wrote the first draft of the manuscript, supervised the preparation of the manuscript, and contributed intellectual and practical work to the manuscript. MB edited the draft, revised it critically, and contributed intellectually and practically to the manuscript. Both authors finally approved the manuscript. Both authors are accountable for all aspects of the work.

## Conflict of Interest Statement

The authors declare that the research was conducted in the absence of any commercial or financial relationships that could be construed as a potential conflict of interest.

## Appendix

**Table A1:** Model summary of the hierarchical regression analysis examining interaction effects between the GDT version (group), executive functions (MCST errors), and ANP skills (number-comparison accuracy) on risky decision making in the GDT (one single digit).

Model	*R*^2^	Δ*R*^2^	Δ*F*	*p*
(1) Group (GDT version)	0.004	0.004	0.55	0.458
(2) MCST (total errors)	0.073	0.068	9.22	0.003
(3) Number-comparison (total accuracy)	0.073	0.001	0.09	0.771
(4) Group × MCST	0.077	0.003	0.46	0.501
(5) Group × Number-comparison	0.080	0.003	0.37	0.545
(6) MCST × Number-comparison	0.093	0.013	1.74	0.189
(7) Group × MCST × Number-comparison	0.093	0.001	0.08	0.779

*GDT, Game of Dice Task; MCST, Modified Card Sorting Test; N = 128.*

**Table A2:** Statistics of the coefficients in the final step of the moderated regression analysis examining interaction effects between the GDT version (group), executive functions (MCST errors), and ANP skills (number-comparison accuracy) on risky decision making in the GDT (one single digit).

Model variables	*b*	*SE*	β	*t*	*p*
**Predictor variables**					
Group (GDT version)	0.18	0.26	0.058	0.69	0.489
MCST (total errors)	0.14	0.04	0.344	3.94***	<0.001
Number-comparison (accuracy at ratio )	–2.19	1.46	–0.126	–1.50	0.136
**2-way interactions**					
Group × MCST	–0.01	0.03	–0.028	–0.32	0.751
Group × Number-comparison	0.61	1.46	0.035	0.42	0.677
MCST × Number-comparison	–0.78	0.21	–0.342	–3.82***	<0.001
**3-way interaction**					
Group × MCST × Number-comparison	0.04	0.21	0.016	0.18	0.857

*GDT, Game of Dice Task; MCST, Modified Card Sorting Test; N = 128. Please note that the positive beta value of MCST errors indicates associations between reduced executive functions (more errors) and reduced GDT performance (more riskiest choices). Conversely, negative betas regarding number-comparison (high accuracy) indicate a positive effect on GDT performance (less risky choices); ^∗^p < 0.05, ^∗∗^p < 0.01, ^∗∗∗^*p* < 0.001.*

**Table A3:** Model summary of the hierarchical regression analysis examining interaction effects between the GDT version (group), objective numeracy (total correct), and ANP skills (number-comparison accuracy) on risky decision making in the GDT (one single digit).

Model	*R*^2^	Δ*R*^2^	Δ*F*	*p*
(1) Group (GDT version)	0.004	0.004	0.55	0.458
(2) Numeracy (total correct)	0.246	0.241	39.95	<0.001
(3) Number-comparison (total accuracy)	0.246	<0.001	0.02	0.891
(4) Group × Numeracy	0.263	0.017	2.87	0.093
(5) Group × Number-comparison	0.271	0.008	1.37	0.244
(6) Numeracy × Number-comparison	0.276	0.005	0.78	0.380
(7) Group × Numeracy × Number-comparison	0.283	0.008	1.28	0.261

*GDT, Game of Dice Task; N = 128.*

**Table A4:** Statistics of the coefficients in the final step of the moderated regression analysis examining interaction effects between the GDT version (group), objective numeracy (total correct), and ANP skills (number-comparison accuracy) on risky decision making in the GDT (one single digit).

Model variables	*b*	*SE*	β	*t*	*p*
**Predictor variables**					
Group (GDT version)	0.13	0.23	0.041	0.55	0.582
Numeracy (total correct)	–0.66	0.12	–0.429	–5.49***	<0.001
Number-comparison (accuracy at ratio )	–1.44	1.33	–0.083	–1.08	0.281
**2-way interactions**					
Group × Numeracy	0.16	0.12	0.100	1.28	0.204
Group × Number-comparison	1.30	1.33	0.075	0.97	0.332
Numeracy × Number-comparison	2.12	0.67	0.248	3.17**	0.002
**3-way interaction**					
Group × Numeracy × Number-comparison	–0.36	0.67	–0.042	–0.53	0.594

*GDT, Game of Dice Task; MCST, Modified Card Sorting Test; N = 128. Please note that the positive beta value of MCST errors indicates associations between reduced executive functions (more errors) and reduced GDT performance (more riskiest choices). Conversely, negative betas regarding number-comparison (high % correct) indicate a positive effect on GDT performance (less risky choices); ^∗^p < 0.05, ^∗∗^p < 0.01, ^∗∗∗^*p* < 0.001.*
